# A New Medical Dictionary

**Published:** 1848-01

**Authors:** 


*A New Medical Dictionary;* containing an explanation of the terms of Anatomy human and comparative, Physiology, Practice of Medicine, Obstetrics, Surgery, Therapeutics, Materia Medica, Pharmacy, Chemistry, Botany, Natural Philosophy. With the formulas of the principal Pharmacopœias, and valuable practical articles on the treatment of disease, on the basis of Hooper and Grant. Adapted to the present state of Science and for the use of Medical Students and the Profession. By D. Pereira Gardner, M. D., Prof. of Chemistry and Med. Jurisprudence in the Philadelphia College of Medicine, &c., &c. New-York, Harper and Brothers; 1847. 8vo.

A good Dictionary, now-a-days, is not only a desideratum, but an indispensable hand-book for the medical student and practitioner. If the above work be as comprehensive in its scope as its title page imports, it is all that can be desired. We cannot profess to have imitated the old lady who perused Johnson's dictionary in course from the beginning to the end; but we have had occasion already to refer to it frequently, and we have thus far found it abundantly adequate to our necessities. We think it may be recommened as one of the best, if not *the* best of similar publications now before the medical public.

